# Non-Tuberculous Mycobacterium Peritoneal Dialysis-Associated Peritonitis: A Case Report of *Mycobacterium neoaurum*

**DOI:** 10.1177/20543581261489228

**Published:** 2026-09-10

**Authors:** Aubrey Maltz, Ali Hajar, Ryan D. Cooper

**Affiliations:** 1 Internal Medicine Resident Trainee, Department of Internal Medicine, 12357 University of Alberta, Edmonton, Alberta, Canada; 2 Department of Nephrology, 12357 University of Alberta, Edmonton, Alberta, Canada; 3 Department of Infectious Diseases, 12357 University of Alberta, Edmonton, Alberta, Canada

**Keywords:** peritoneal dialysis–associated peritonitis, nontuberculous mycobacteria, Mycobacterium neoaurum, culture-negative peritonitis, peritoneal dialysis

## Abstract

Peritoneal dialysis (PD) related peritonitis is a common cause for PD technique failure. In up to 20% of cases conventional cultures are negative, and nontuberculous mycobacteria are rarely identified as a cause. A 60-year-old man treated with peritoneal dialysis as kidney replacement therapy presented to care with two months of malaise, abdominal pain, and cloudy PD effluent. Effluent analysis demonstrated a marked neutrophil-predominant pleocytosis, but no organisms were identified on Gram stain or on initial cultures after 72 hours of incubation. He was admitted to the hospital and intraperitoneal vancomycin was initiated for presumed PD–associated peritonitis. After five days, his symptoms and neutrophil-predominant effluent pleocytosis persisted. Repeat effluent Gram stain identified a beaded, Gram-positive bacilli. Subsequent auramine rhodamine stain revealed acid-fast bacilli, ultimately speciating to *Mycobacterium neoaurum*; initial cultures ultimately demonstrated the same bacterium. Intraperitoneal vancomycin was changed to oral doxycycline and moxifloxacin, the PD catheter was removed, and the patient initiated intermittent hemodialysis. There was gradual improvement in presenting symptoms after changing antimicrobials. He was discharged on a 6-month course of doxycycline and moxifloxacin, with repeat clinic visits over 10 months showing resolution of weakness, increase in dry body weight, and no evidence of recurrent disease on cross-sectional imaging. Non-tuberculous mycobacteria PD peritonitis should be considered in patients with a subacute clinical course, initially negative effluent cultures, and lack of response to conventional antimicrobials. *M. neoaurum* has only rarely been associated with PD peritonitis; this case contributes to the evidence of this pathogen’s association with implanted devices.

## Introduction

Peritoneal dialysis (PD) is an important modality of kidney replacement therapy worldwide and offers autonomy through home-based care.^1^ PD-related infections are a common complication of PD and can necessitate catheter removal, temporary transition to hemodialysis, or permanent technique failure. While the majority of cases of PD-associated peritonitis are caused by conventional bacterial pathogens, up to 20% cases are initially culture negative, where no causative organism is identified after 72 hours of incubation.^2^ Culture negative PD-associated peritonitis can be caused by previous antibiotic exposure reducing yield of diagnostic testing, PD catheter-associated biofilm, fungal peritonitis, and nontuberculous mycobacteria (NTM).^3^

NTM includes all mycobacteria besides *M. tuberculosis* and *M. leprae.* Included within this group of mycobacteria are rapidly-growing mycobacteria (RGM), mycobacteria that grow on solid media within 7 days. RGM are commonly found in soil, water, and dust.^4^ *Mycobacterium neoarum* is an RGM historically considered to affect immunocompromised people.^5^ A 2023 review of this isolate found that most reported cases were nosocomial and often device-associated, which often require removal as a part of management.^6^

NTM PD-associated peritonitis is considered a rare entity, and the diagnosis is frequently delayed. Although *M. abscessus, M. fortuitum* and *M. chelonae* infectious have been previously reported in case series,^7-9^ *Mycobacterium neoaurum* is very rarely reported in association with PD-related peritonitis.^5,10^ NTM PD peritonitis introduces substantial morbidity and frequently necessitates PD catheter removal, change of dialysis modality, and treatment with prolonged courses of multiple antibiotics.^8^ Here, we describe a case of *M. neoaurum* causing clinically significant PD-associated peritonitis leading to PD technique failure.

## Presenting Concerns

A 60-year-old man treated with peritoneal dialysis as kidney replacement therapy presented to a Canadian tertiary care centre with two months of progressive weakness, presyncope, and abdominal pain. He reported persistently low systolic blood pressures of 70-80 mmHg despite discontinuing the majority of his home antihypertensives. He noted his peritoneal dialysis fluid had been clear without any pain, erythema or discharge at the catheter exit site.

### Clinical Findings

Past medical history included kidney failure related to longstanding diabetes, for which he received PD as kidney replacement therapy for the past three months. He also had coronary artery disease with ischemic cardiomyopathy and type 1 diabetes mellitus. Family history was noncontributory, he had no known tuberculosis exposures, and he was independent in activities of daily living. His home medications included acetylsalicylic acid, calcitriol, metoprolol, fludrocortisone, and scheduled subcutaneous insulin.

On examination, he appeared slightly drowsy although orientated. He was afebrile, with a supine blood pressure of 103/66 mmHg after intravenous fluid resuscitation; vital signs were otherwise within normal limits. The peritoneal dialysis catheter exit site was clean without surrounding erythema, induration, or drainage. Abdomen was soft, moderately distended, and although slightly tender to deep palpation, there were no peritoneal signs.

Initial hemoglobin was 112 g/L, white blood cell count was 10.0 ×10^9^/L with a neutrophilic predominance (9.3 ×10^9^/L), and platelet count was 377 ×10^9^/L. Electrolytes were unremarkable and his creatinine was 730 µmol/L, near his baseline. Conventional blood cultures were negative. Initial urinalysis demonstrated mild proteinuria and glycosuria without nitrites, leukocytes, or bacteria. Alkaline phosphatase was elevated at 290 U/L (reference 40-120), near his prehospital baseline; other liver enzymes, liver function tests, and lipase were normal. C-reactive protein was elevated at 108.0 mg/dL (reference < 8.0). A chest x-ray showed a subsegmental atelectasis in the left lung with no other acute changes noted. Hemoglobin A1c was 9.8%.

Visibly cloudy PD dialysis effluent was sampled on the first day after admission, despite the patient not recalling cloudy effluent prehospital. PD effluent cell count was 5100 cells/μL, of which 99% were neutrophils, consistent with PD-associated peritonitis, however his gram stain demonstrated no bacteria.

### Timeline

Table 1 documents the patient’s clinical course.

**Table 1.** Clinical Course Timeline

Time point	Clinical status
12 months prior to admission	Peritoneal dialysis catheter inserted
3 months prior to admission	PD catheter exteriorized; patient initiated on PD
2 months prior to admission	Development of progressive hypotension, malaise, and weight loss
Date of admission	Patient presented with progressive symptoms and was found to have cloudy PD effluent with neutrophilic pleocytosis (5,100 cells/µL) with negative gram stain. Intraperitoneal vancomycin and prophylactic swish and swallow oral liquid nystatin initiated.
Post-admission day 4	Persistent malaise and abdominal pain with ongoing PD effluent neutrophilic pleocytosis (1,700 cells/µL). Previously obtained peritoneal fluid cultures demonstrated acid-fast bacilli on broth culture and Gram-positive bacilli on separate cultures.
Post-admission day 7	PD effluent cell count remained elevated at 300 cells/µL. Acid-fast bacilli speciated as *Mycobacterium neoaurum*. CT abdomen and pelvis with intravenous contrast demonstrated mildly enlarged cystic and necrotic-appearing inguinal lymph nodes.
Post-admission day 10	Repeat PD effluent culture obtained redemonstrated acid-fast bacilli. Antimicrobials were changed to doxycycline 100 mg orally twice daily and moxifloxacin 400 mg orally daily; intraperitoneal vancomycin and Nystatin oral liquid discontinued.
Post-admission day 12	Tunneled right internal jugular hemodialysis catheter inserted
Post-admission day 13	Peritoneal dialysis catheter removed with surgical debridement of abdominal wall, transitioned to intermittent hemodialysis
Post-admission day 15	Patient doing well, tolerating antimicrobials well, discharged from hospital. *M. neoaurum* drug susceptibilities reported and include susceptibility to doxycycline and moxifloxacin.
2 months - 6 months post-admission	Patient tolerated antimicrobial therapy and completed a six-month course of doxycycline and moxifloxacin
8 months post admission	CT abdomen and pelvis with intravenous contrast demonstrated improvement in cystic and necrotic inguinal lymphadenopathy, with no evidence of ongoing infection

### Diagnostic Focus and Assessment

He was admitted to the kidney medicine service for further evaluation and management hypotension and PD-associated peritonitis and was started on empiric intraperitoneal vancomycin at a dose of 2 g every five days, as well as nystatin oral liquid swish and swallow 500,000 units four times daily as fungal peritonitis prophylaxis. The admitting team did not feel it was necessary to cover for gram negatives, as the initial gram stain revealed no organisms.

Four days into admission, the PD effluent neutrophil count was still significantly elevated at 1,700 cells/µL and malaise had not resolved. This incomplete response to empiric therapy raised a broader differential diagnosis including infection with a drug-resistant organism or secondary peritonitis.

A contrast-enhanced CT scan of the abdomen and pelvis demonstrated no radiographic evidence of secondary peritonitis. The scan did reveal enlarged necrotic-appearing inguinal lymph nodes of unclear relevance. No previous abdominal imaging was available for comparison.

Gram staining of initial peritoneal fluid effluent samples did not reveal any microorganisms and bacterial cultures remained negative over the first 72 hours of incubation.

The lack of microbiologic confirmation, combined with lack of response to empiric antibacterial therapy prompted concern of infection with atypical organisms. Repeat samples of peritoneal fluid were thus submitted for bacterial, mycobacterial, and fungal culture. After 4 days of incubation, multiple specimens were found culture positive for acid-fast bacilli, eventually identified as M. neoaurum using MALDI-TOF. The acid-fast bacilli prompted infectious diseases consultation.

### Therapeutic Focus and Assessment

Given the persistently elevated neutrophil count in the peritoneal dialysis effluent and evidence of non-tuberculous mycobacterial growth, intraperitoneal vancomycin and nystatin were discontinued and doxycycline 100 mg orally twice daily and moxifloxacin 400 mg orally daily commenced. A combination regimen was selected to reduce the risk of antimicrobial resistance, a well-recognized cause of treatment failure in nontuberculous mycobacterial infection.^1,9^

He was started on intermittent hemodialysis with a newly placed tunneled central venous catheter. The peritoneal dialysis catheter was completely removed. Over the coming weeks, the patient reported improved abdominal pain and malaise and he was discharged home on intermittent hemodialysis.

### Follow-Up and Outcomes

In the ambulatory setting, the patient eventually completed a 6 month course of doxycycline and moxifloxacin. The systemic antibiotic therapy was tolerated well. He also gained weight and noticed improved functional status. Repeat cross-sectional imaging showed the asymptomatic inguinal lymphadenopathy was unchanged. Presently, he is living independently and undergoing intermittent hemodialysis; he is considering pursuing kidney transplantation.

## Discussion

While PD-associated peritonitis is usually caused by aerobic Gram positive and facultative Gram-negative bacteria, clinical failure with empiric broad-spectrum antibiotics and negative routine cultures should raise suspicion for non-tuberculous mycobacterial peritonitis. In this case, persistent peritonitis refractory to empiric intra-peritoneal antibacterial therapy, a subacute presentation, and lack of CT abdomen findings of secondary peritonitis raised suspicion for NTM PD-associated peritonitis, later confirmed by mycobacterial growth from PD effluent.

This case shares key features with prior reports of *M. neoaurum* PD-associated peritonitis, including persistent abdominal pain and elevated effluent neutrophil counts despite empiric therapy. Similarly, McNally et al. described a subacute presentation refractory to standard treatment with vancomycin and gentamicin which ultimately required PD catheter removal.^5^ Management was distinct, with them ultimately describing a course of 4 months of ciprofloxacin and rifampin with a 1 month gentamicin. An Australian case series described similar initial empiric therapy (vancomycin, gentamicin) followed by ciprofloxacin and amikacin for an unclear duration, but with PD catheter retention.^10^ These cases highlight a subacute, treatment-refractory presentation, with key differences in antimicrobial strategy and PD catheter management.

This case report does have limitations. The admitting team’s initial intraperitoneal antimicrobial did not cover for gram-negatives, which are recommended by ISPD guidelines for empiric treatment of PD-associated peritonitis pending the availability of PD effluent culture results to guide definitive therapy.^1^ The presence of inguinal lymphadenopathy was a somewhat nonspecific finding, and is not classically associated with NTM PD-associated peritonitis but could have been biopsied to further substantiate treatment. There is also limited data to substantiate choice and duration of antimicrobial therapy in NTM PD-associated peritonitis.^1,9^

NTM PD-associated peritonitis can be a challenging diagnosis to recognize due to low index of suspicion given its rarity.^1,3,9^ Conventional culture methods, although fortuitously positive in this case, typically have poor sensitivity for detection of NTM in peritoneal effluent. Thus, unless samples specifically submitted in appropriate mycobacterial transport and growth media, the diagnosis will not be confirmed. Even with appropriate sample collection, prolonged time to positivity can contribute to diagnostic delay. NTM PD-associated peritonitis should be suspected in patients with slow or inadequate response to conventional treatment: consultation with the microbiology team may be needed for expedited evaluation. RGMs like *M. neoaurum* are associated with biofilm formation, often necessitating the removal of any implanted devices.^5,6^ This case also contributes to the growing body of evidence that *M. neoaurum* is a healthcare and device-associated pathogen.
